# Secondary consent to biospecimen use in a prostate cancer biorepository

**DOI:** 10.1186/s13104-016-2159-3

**Published:** 2016-07-18

**Authors:** Bettina F. Drake, Katherine Brown, Lucy D’Agostino McGowan, Jennifer Haslag-Minoff, Kimberly Kaphingst

**Affiliations:** Division of Public Health Sciences, Washington University School of Medicine, 600 S. Taylor Ave, Campus Box 8100, St. Louis, MO 63110 USA; Alvin J. Siteman Cancer Center, St. Louis, MO USA; Vanderbilt University, Nashville, TN USA; University of Missouri School of Medicine, Columbia, MO USA; Department of Communication, University of Utah, Salt Lake City, UT USA; Huntsman Cancer Institute, Salt Lake City, UT USA

**Keywords:** Biorepository, Protected health information, Consent, African–American, Prostate cancer

## Abstract

**Background:**

Biorepository research has substantial societal benefits. This is one of the few studies to focus on male willingness to allow future research use of biospecimens.

**Methods:**

This study analyzed the future research consent questions from a prostate cancer biorepository study (N = 1931). The consent form asked two questions regarding use of samples in future studies (1) without and (2) with protected health information (PHI). Yes to both questions of use of samples was categorized as Yes-Always; Yes to without and No to with PHI was categorized as Yes-Conditional; No to without PHI was categorized as Never. We analyzed this outcome to determine significant predictors for consent to Yes-Always vs. Yes-Conditional.

**Results:**

99.33 % consented to future use of samples; 88.19 % consented to future use without PHI, and among those men 10.2 % consented to future use with PHI. Comparing Yes Always and Yes Conditional responses, bivariate analyses showed that race, family history, stage of cancer, and grade of cancer (Gleason), were significant at the α = 0.05 level. Using stepwise multivariable logistic regression, we found that African–American men were significantly more likely to respond Yes Always when compared to White men (p < 0.001). Those with a family history of prostate cancer were significantly more likely to respond Yes Always (p = 0.002).

**Conclusions:**

There is general willingness to consent to future use of specimens without PHI among men.

## Background

Research with stored biospecimens has generated extensive clinical data essential for the advancement of translational medicine [1–7]. Such research can provide critical societal benefits, including enhanced medical treatments and improved national health outcomes [5, 6, 8]. However, the question of patient donation of biospecimens for unplanned secondary research purposes raises important ethical considerations [4–7]. Biospecimen donors who consent to unspecified future research uses are vulnerable to protected health information (PHI) disclosure, loss of control, and risk of exploitation [3–7]. However, regulations require researchers to be diligent in the security and safety of all data, specifically, PHI on participants. Under the Health Insurance Portability and Accountability Act (HIPAA) PHI includes social security and medical record numbers, names, zip codes, dates of birth, contact information including telephone, fax, email and mailing addresses. The views and willingness of participants to provide PHI along with samples and health information is important to fully explore. The Department of Health and Human Services is currently revising the ‘Common Rule’ to require a broad informed consent for secondary research with a biospecimen even if PHI is not being provided [9]. Research on individuals’ attitudes and willingness to donate biospecimens for unplanned future uses are essential for informing these ethical concerns [4–7]. It is important to further recognize the lack of information surrounding biorepository participation and patient attitudes and willingness to donate among diverse sociodemographic groups.

These issues are particularly salient in the context of cancer. Research with stored biospecimens can provide a greater understanding of cancer etiology and discovery of new therapeutic modalities [4–7]. Participation in biobanks by large numbers of diverse participants is critical to reaching translational research goals in cancer and reducing cancer disparities [8]. However, participants who donate biospecimens for research may face risks related to unwanted disclosure of PHI which may affect willingness to participate among diverse individuals [10, 11]. A disproportionate burden of cancer disparities is borne by communities increasingly of interest to biobank researchers but few studies have examined the consent preferences and needs of underserved communities [12, 13].

In order to gain further insight into these attitudes towards secondary biospecimen usage, research must include underrepresented minorities and groups [3, 6, 8, 14]. One group that has been particularly underrepresented in this type of research is males, specifically African American men. The proposed study responds to this gap in the literature by including African American male prostate cancer patients and investigating their willingness to participate in secondary consent to research use of biospecimens.

## Methods

### Study population

This study analyzed secondary questions added to a consent form regarding consent for future research uses of biospecimens. The primary consent was for participation in a prostate cancer biorepository study (N = 1931) among White and African American (12.3 %) men. The prostate cancer biorepository study enrolled men after a prostate cancer diagnosis but before treatment to collect serum and tissue for analyses on genetic, dietary and clinical risk factors for prostate cancer recurrence and mortality. Participants consented to participate in a study that will assess genetic risk for prostate cancer outcomes. At enrollment, a detailed chart review is performed to document the patient’s medical history as well as the clinical characteristics of their cancer diagnosis and treatment. The Washington University in St. Louis Institutional Review Board approved this study and consent procedures.

### Variable definition

**Fig. 1:**
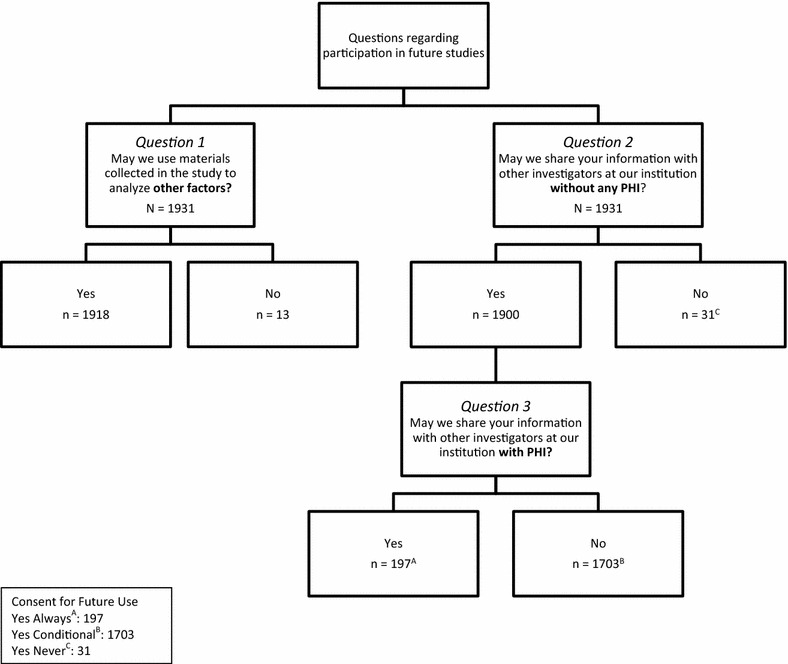
Consent for future use definition

On the consent form, men were asked three separate questions regarding participation in future studies: (1) May we use the materials collected in this study to analyze other factors? Other factors may include analyses, other than genetic, of markers derived from stored serum which were not part of the original research question. (2) May we share your information with other investigators at our institution without any PHI? (3) May we share your information with other investigators at our institution with PHI? All men were asked questions 1 and 2. Only men who responded yes to question 2 (May we share your information with other investigators at our institution without any PHI?) were asked question 3 regarding use of information with PHI. If a participant responded No to question 2 (use without PHI), they were not asked question 3 (use with PHI) (see Fig. 1). Men who responded Yes to question 2 (use without PHI) and Yes to question 3 (use with PHI) were categorized as Yes Always consent for future use. Men who responded Yes to question 2 (use without PHI) and No to question 3 (use with PHI) were categorized as Yes Conditional consent for future use. Men who responded No to question 2 (use without PHI) were categorized as Never consent for future use.

Predictors of consent for future use were obtained from the baseline survey and clinical data obtained from participants in the cohort at the time of diagnosis. Variables assessed were race, age, marital status, employment status, smoking history, family history of prostate cancer, stage of cancer, and grade of cancer.

### Statistical analysis

Statistical analyses were performed by using SAS/STAT^®^ 9.4 (SAS Institute, Cary, NC). Because so few patients were in the “Never” group, we limited the bivariate and multivariate analyses to only participants in the “Yes Always” and “Yes Conditional” groups. Since very few participants were in the “Other” race category, we limited the analysis to African American/Black and White participants. We performed bivariate analyses using Chi squared tests for categorical variables (race, marital status, employment status, smoking history, family history of prostate cancer, stage of cancer, and grade of cancer) or Fisher’s exact test for variables with expected cell counts less than 5 and a student’s *t* test for the continuous variable, age. Multivariable logistic regression analyses were used to identify factors associated with consent for future use responses. In these models, age was continuous, and the remaining variables, race, marital status, employment status, smoking history, family history of prostate cancer, stage of cancer, and grade of cancer (Gleason), were categorical. We employed stepwise selection with entry criteria set at 0.1 and the cutoff of 0.05 to remain in the model. Participants were excluded if they had missing data for any of the variables included in the model.

## Results

There were 1931 participants who responded to the secondary consent questions. 99 % consented to future use for other factors; 88 % consented to future use with other investigators without any PHI, and among those men 10.2 % consented to future use with other investigators with PHI. Descriptive statistics of the participants are reported in Table 1.

**Table 1:** Descriptive characteristics

	N	n	(%)
Outcomes			
Does patient agree to allow study team to analyze other factors?	1931		
Yes		1918	(99.33)
No		13	(0.67)
Consent for future use	1931		
Yes always		197	(10.2)
Yes conditional		1703	(88.19)
Never		31	(1.61)
Predictors			
Age at diagnosis	1859		
Mean (SD)		61.03	(7.41)
Race	1931		
White		1682	(87.11)
Black		237	(12.27)
Other		12	(0.62)
Family history of prostate cancer	1733		
Yes		615	(35.49)
No		1118	(64.51)
Marital status	1322		
Married		1133	(85.70)
Separated/divorced/widowed		155	(11.72)
Never married		34	(2.57)
Employment status	1312		
Employed		712	(54.27)
Retired		530	(40.40)
Unemployed		47	(3.58)
Other		23	(1.75)
Smoking status	1889		
Never smoked		918	(48.60)
Former smoker		753	(39.86)
Current smoker		218	(11.54)
Clinical stage	1881		
T1c		1512	(80.38)
T2a/T2b/T2c		369	(19.62)
Gleason score	1888		
2–6		1147	(60.75)
7		587	(31.09)
8+		154	(8.16)

**Fig. 2:**
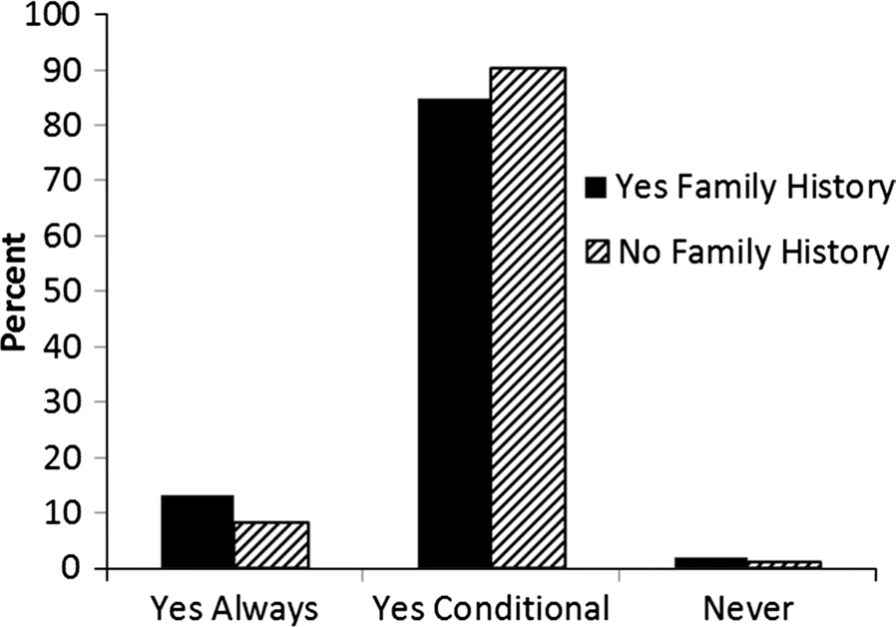
Secondary consent by family history of prostate cancer

**Fig. 3:**
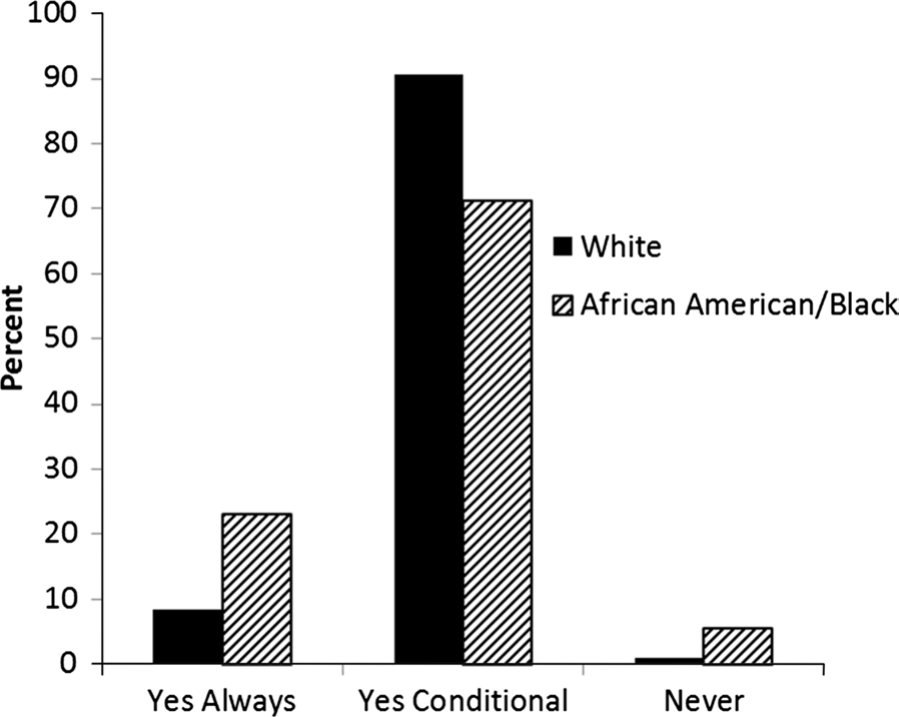
Secondary consent by race

Figure 2 displays the percent of consent for future use option stratified by family history of prostate cancer. This chart shows that 13 % of those who responded “Yes” to having a family history of prostate cancer responded “Yes Always” as compared to 8 % of those who responded “No” to having a family history of prostate cancer. Figure 3 displays the percent of consent for future use option stratified by race. From this chart, we can see that 23 % of African American/Black participants responded “Yes Always”, as compared to 8 % of White participants that responded the same.

Of the 1931 participants, 1900 responded “Yes Always” or “Yes Conditional” to consent to future use. Due to the small number of participants that responded “Never” (N = 31) and the small number of participants who indicated that they identified as a race other than African American/Black or White (N = 12), we excluded these participants from the remainder of our analyses, leaving 1889 participants. Table 2 shows the prevalence of response type by age, race, marital status, employment status, smoking history, family history of prostate cancer, stage of cancer, and grade of cancer. The variables race, family history, stage of cancer, and grade of cancer were significantly associated with consent for future use when analyzed alone. Black participants were 3.49 times more likely to agree to consent for future use “Always” when compared to the White participants (p < 0.001). Those with a family history of prostate cancer were 1.69 times more likely to agree to consent to future use “Always” when compared to those without a family history (p = 0.001). Prostate cancer is defined as clinical stage 1 if it is detected by PSA screening only and is not detectable by imaging. As noted in Table 1, most men in our sample are classified as Clinical Stage T1c, meaning their prostate cancer was identified by needle biopsy. Prostate cancer is defined as clinical tumor stage T2 if there is a tumor present, and it is confined within the prostate [15]. Participants with clinical tumor stage T2 cancer were less likely to agree to consent to future use “Always” compared to men with clinical tumor stage T1 cancer (OR: 0.52, p = 0.005). However, men with a Gleason score of 7 were 1.93 times more likely to agree to consent to future use “Always” than men with lower Gleason scores (p < 0.001).

**Table 2:** Bivariate associations between sociodemographic and clinical predictors and willingness to donate biospecimens for future research uses (N = 1889)

	N	Yes conditional **N (%)**	Yes always **N (%)**	χ2	OR (95 % CI) always (vs. conditional)	p value
Age at diagnosis	1859					
Mean (SD)		61.0 (7.4)	61.4 (7.2)	−0.75^a^	–	0.453
Race	1889			54.28		<0.001*
White		1523 (90.0)	142 (72.1)		1.00 (ref)	
Black		169 (10.0)	55 (27.9)		3.49 (2.46, 4.95)	
Family history	1694			10.53		0.001*
No		1004 (66.0)	92 (53.5)		1.00 (ref)	
Yes		518 (34.0)	80 (46.5)		1.69 (1.23, 2.32)	
Marital status	1301					0.415^b^
Married		1049 (86.0)	66 (81.5)		1.00 (ref)	
Not currently married		141 (11.6)	12 (14.8)		1.35 (0.71, 2.57)	
Never married		30 (2.5)	3 (3.7)		1.59 (0.47, 5.34)	
Employment status	1292					0.104^b^
Employed		661 (54.6)	40 (49.4)		1.00 (ref)	
Retired		489 (40.4)	32 (39.5)		1.08 (0.67, 1.75)	
Unemployed		41 (3.4)	6 (7.1)		2.42 (0.97, 6.04)	
Other		20 (1.7)	3 (3.7)		2.48 (0.71, 8.70)	
Smoking	1847			0.34		0.844
Never smoked		800 (48.4)	98 (50.2)		1.00 (ref)	
Former smoker		660 (39.9)	75 (38.7)		0.93 (0.68, 1.28)	
Current smoker		193 (11.7)	21 (10.8)		0.89 (0.54, 1.46)	
Stage	1841			7.78		0.005*
T1c		1316 (79.4)	162 (88.0)		1.00 (ref)	
T2a/T2b/T2c		341 (20.6)	22 (12.0)		0.52 (0.33, 0.83)	
Gleason score	1846			17.22		<0.001*
2–6		1035 (62.5)	89 (47.1)		1.00 (ref)	
7		489 (29.5)	81 (42.9)		1.93 (1.40, 2.65)	
8+		133 (8.0)	19 (10.1)		1.66 (0.98, 2.82)	

* Significant at the α = 0.05 level

^a^t value is calculated for continuous variables

^b^Fisher’s exact test is used for variables with expected cell counts <5

In a combined model, race and family history of prostate cancer were significant with an alpha = 0.1 criteria for entry into the model and alpha = 0.05 criteria to remain in the model. There were 1694 participants included in our final model. Of these, 1522 (89.8 %) responded “Conditional” to consent to future use, and 172 (10.2 %) responded “Always”. The final prediction model with race and family history can be seen in Table 3. In this combined model, Black participants were 3.17 times more likely to agree to consent for future use “Always” (p < 0.001); likewise, men with a family history of prostate cancer were 1.67 times more likely to agree to consent for future use “Always” (p = 0.002).

**Table 3:** Logistic regression predicting willingness to donate biospecimens for future research uses (N = 1694)

Effect	OR	95 % CI		p value
Odds ratio estimates always (vs. conditional)				
Race				<0.001
White	1.00 (ref)	–	–	
Black	3.17	2.15	4.67	
Family history				0.002
No	1.00 (ref)	–	–	
Yes	1.67	1.21	2.31	

## Discussion

There is a general willingness to consent to secondary research use of biospecimens among men without PHI. While African American men are more likely to consent to future research “Never” than men of other racial backgrounds, those who do agree are more likely to say “Yes Always” to research with and without PHI when compared to the White population. Additionally, those with a family history are more likely to agree to secondary uses with PHI than those without a family history as compared to agreeing to secondary use only without PHI.

Past literature has investigated the attitudes of females regarding initial and secondary usage of biospecimens [1–5, 7]. Female participation in biospecimen research has reflected high rates of willingness and consent for unlimited future research [4, 7]. Of those studies that included males, a number of studies have found that there was no significant difference between sexes when measuring willingness to participate in secondary research use of biospecimens [1, 5, 7]. Studies that further compared healthy male and female volunteers to diseased individuals also did not find any significant difference in consent for future use of biospecimens [3, 7]. One past research study that exclusively surveyed male cancer patients showed 100 % consent rate to participate in biobank donation [6]. However, few studies have focused only on males. In addition, our study assessed actual donors’ consent and not just willingness to consent. Past research has also found significant differences in consent for future uses of secondary biospecimens between races, particularly comparing Caucasians and African Americans [1, 5, 7]. Some studies found no significant difference in consent for biospecimen studies [16, 17], while another study found African–Americans were less likely but with a high percentage of 75 % African Americans consenting to a biospecimen study [18]. Our study focuses on consent to secondary use of biospecimens and only focuses on men which have not been thoroughly evaluated in previous studies.

Despite the large percentage of men who agreed to future use of their biospecimens in our study, many did not consent to sharing their PHI in future research projects. Usability of de-identified biospecimens differs from those with identifiers attached. Many pathology departments have developed honest broker services which provide a firewall between clinical records and research studies. The honest broker provides de-identified clinical data to researchers for research use only [19]. However in the absence of an honest broker, participants must provide consent for secondary use of their biospecimens. However, data from de-identified biospecimens may still be analyzed and utilized by clinical investigators; several studies using de-identified samples have been able to confirm and replicate previous findings [20, 21].

This study had a few limitations. In order to participate in the initial study, participants had to agree to genetic analyses; therefore we are unable to determine if preferences for secondary genetic research would be similar to the preferences for secondary research with vs. without PHI described here. In addition, of the 31 who were categorized as consenting to future use “Never”, only 12 responded No to the initial question, “May we use the materials collected in this study to analyze other factors?” The other 19 men agreed to allow the study team to analyze other factors but under no circumstances (with or without PHI). This may speak to some confusion in the consent process among participants when they respond yes or no to allow researchers to analyze other factors. More African American men chose the extremes of Yes Always or Never.

Future research should focus on informed decision making studies to assess whether these choices are in alignment with their values and preferences. In addition, studies are needed to examine whether more focused education about the protections in place for PHI would encourage unrestricted donation of biospecimens.

## Conclusions

It is critical for researchers to understand patient perceptions and needs of all population subgroups in order to provide fair and just treatment regarding biospecimen collection practices and to improve informed decision making about biospecimen donation. Based on our findings, there is general willingness to consent to future use of specimens without PHI among men. We found there were differences by race in consent to secondary use of biospecimens. Inclusion of underrepresented populations in biospecimen research is critically needed to enhance diversity within biobanks so that results generated by biobank research are applicable to all segments of the population. Otherwise, the societal benefits of translational research using biospecimens are limited. The current study is the first to investigate the willingness and participation extent in secondary biospecimen research in an underrepresented group, African American men. This study can inform consent protocols and educational needs for enrolling male patients from different population groups into biobanks.

## Availability of data and materials

The data is not deposited in a publicly available repository.

## Abbreviation

PHI: protected health information

## References

[1] Scott EA (2010). Biospecimen repositories: are blood donors willing to participate?. Transfusion.

[2] Master Z, et al. Cancer patient perceptions on the ethical and legal issues related to biobanking. BMC Med Genomics. 2013; 6(8). 10.1186/1755-8794-6-8 PMC3599691 23497701

[3] Malone T (2002). High rate of consent to bank biologic samples for future research: the Eastern Cooperative Oncology Group experience. J Natl Cancer Inst.

[4] Helft PR, et al. Cancer patients’ attitudes toward future research uses of stored human biological materials. 2007. 10.1525/jer.2007.2.3.15 19385847

[5] Pentz RD, Billot L, Wendler D (2006). Research on stored biological samples: views of African American and White American cancer patients. Am J Med Genet Part A.

[6] Huber J, et al. Two decades’ experience with a prospective biobank for urologic oncology: research, clinical care, and the patients’ view. In: Urologic Oncology: Seminars and Original Investigations. 2012. Elsevier. 10.1016/j.urolonc.2012.01.016 22386623

[7] Chen DT (2005). Research with stored biological samples: what do research participants want?. Arch Intern Med.

[8] Luque JS (2012). Formative research on perceptions of biobanking: what community members think. J Cancer Educ.

[9] Services’, U.D.o.H.a.H. Federal policy for the protection of human subjects. docket number: HHS-OPHS-2015-0008. Federal Register, 2015; 80(173).

[10] Saha K, Hurlbut JB (2011). Research ethics: treat donors as partners in biobank research. Nature.

[11] Caulfield T, Upshur RE, Daar A (2003). DNA databanks and consent: a suggested policy option involving an authorization model. BMC Med Ethics.

[12] Rennie S (2011). In whose interests?. Hastings Cent Rep.

[13] Trinidad SB, FS, Ludman EJ, Jarvik GP, Larson EB, Burke W. The risks and benefits of reconsent: response. Science. 2011; 332(306).

[14] Murphy J (2009). Public perspectives on informed consent for biobanking. Am J Public Health.

[15] Society. A.C. How is prostate cancer diagnosed? 2014. http://www.cancer.org/cancer/prostatecancer/detailedguide/prostate-cancer-diagnosis.

[16] Scott EA (2010). Biospecimen repositories: are blood donors willing to participate?. Transfusion.

[17] Pentz RD, Billot L, Wendler D (2006). Research on stored biological samples: views of African American and White American cancer patients. Am J Med Genet A.

[18] Chen DT (2005). Research with stored biological samples: what do research participants want?. Arch Intern Med.

[19] Dhir R (2008). A multidisciplinary approach to honest broker services for tissue banks and clinical data: a pragmatic and practical model. Cancer.

[20] Ritchie MD (2010). Robust replication of genotype-phenotype associations across multiple diseases in an electronic medical record. Am J Hum Genet.

[21] Birdwell KA (2012). The use of a DNA biobank linked to electronic medical records to characterize pharmacogenomic predictors of tacrolimus dose requirement in kidney transplant recipients. Pharmacogenet Genomics.

